# Feasibility and Safety of the Early Introduction of Allergenic Foods in Asian Infants with Eczema

**DOI:** 10.3390/nu16111578

**Published:** 2024-05-23

**Authors:** Daisuke Harama, Mayako Saito-Abe, Sayaka Hamaguchi, Tatsuki Fukuie, Yukihiro Ohya, Kiwako Yamamoto-Hanada

**Affiliations:** 1 Allergy Center, National Center for Child Health and Development, 2-10-1 Okura, Setagaya-ku, Tokyo 157-8535, Japan; harama-d@ncchd.go.jp (D.H.); saito-myk@ncchd.go.jp (M.S.-A.); hamaguchi-sa@ncchd.go.jp (S.H.); fukuie-t@ncchd.go.jp (T.F.); ohya-y@ncchd.go.jp (Y.O.); 2 Medical Support Center for the Japan Environment and Children’s Study, National Research Institute for Child Health and Development, 2-10-1 Okura, Setagaya-ku, Tokyo 157-8535, Japan; 3 Department of Pediatrics, Tokyo Metropolitan Hiroo Hospital, 2-34-10 Ebisu, Shibuya-ku, Tokyo 150-0013, Japan

**Keywords:** food allergy, early introduction, infant, eczema

## Abstract

Background: There is a lack of data regarding the early introduction of the consumption of allergenic food among Asian infants. Methods: We examined infants who had early-onset eczema before 6 months of age and received instructions from certified allergists for the early introduction of hen’s eggs, milk, wheat, peanuts, and tree nuts. Results: The consumption rates of hen’s eggs were 100% at 24 months. For peanuts and walnuts, the consumption rate was moderate at 12 months (48.5% and 30.3%, respectively), but by 24 months, it had progressed to 78.8% and 81.3%, respectively. In contrast, cashews remained at lower levels than other allergens at 20.7% at 12 months and 41.4% at 24 months. No adverse events related to early introductions occurred. Conclusions: In infants with eczema, allergenic foods could be introduced early and well tolerated in Asian infants. However, having eczema may indicate a predisposition to food allergies, so caution is necessary when introducing allergenic foods. The early introduction of peanuts and tree nuts was still more challenging in real-world practice in Asia as well as in Western countries.

## 1. Introduction

Peanut and tree nut allergies are prevalent worldwide in recent years. Although regional variations exist, peanut and tree nut allergies are associated with a high rate of anaphylaxis and represent a significant societal burden [1]. Presently, strategies to manage food allergies primarily revolve around avoiding allergenic foods. Regarding treatments, oral immunotherapy for peanut allergies has shown effectiveness; however, the incidence of adverse reactions remains considerable, casting doubts on its safety [2]. There is a notable scarcity of evidence supporting established treatments for tree nut allergies, underscoring a critical area for research. Preventing food allergies in infants and toddlers at risk is paramount. Evidence from randomized controlled trials suggests that the early introduction of foods such as hen’s eggs, peanuts, and cow’s milk can reduce the prevalence of food allergies [3,4,5]. In addition, the early intake of multiple foods (not including nuts) concomitantly has been reported to prevent the development of allergy to those foods [6,7], and a systematic review also considered the early intake of allergenic foods to be promising [8]. On the other hand, these reports do not include tree nuts, and the existing evidence regarding the efficacy and safety of early introduction of tree nuts remains insufficient.

A systematic review addressed insufficient evidence of the timing of the introduction of any solid food for the risk of eczema [9]. However, past studies showed that eczema is a major risk factor for food allergy. Infants with eczema were 11.0 times more likely to develop a peanut allergy and 5.8 times more likely to develop an allergy to hen’s eggs by 12 months than infants without eczema [10]. In particular, for infants within the first 0–3 months after birth, an onset of eczema was found to be associated with an increased risk of developing food allergy at 2 years of age [11,12,13]. It is crucial for the prevention strategy of food allergies to safely introduce allergenic foods appropriately and early in high-risk groups such as eczema infants rather than in the general population [14,15]. In Japan, allergies to hen’s eggs are the most common in infants. However, the prevalence of nut allergies is rapidly increasing. Among nut allergies, a walnut allergy accounts for the highest incidence, followed by a cashew allergy [16]. On the other hand, in traditional Japanese cuisine, the use of nuts is rare, and there is no custom of incorporating nuts into weaning foods from infancy. Moreover, the Japan Pediatric Society and the Japanese Consumer Affairs Agency prohibit the intake of peanuts and hard beans to children under five years of age because of the risk of aspiration and choking. Hence, it is expected that the consumption of peanuts and nuts might be delayed among toddlers in Japan. In consideration of peanut and nut allergy epidemics, we have instructed the early introduction of tree nuts to children as well as hen’s eggs, milk, and peanuts for infants with eczema on a daily basis. There is a lack of data regarding the early consumption of allergenic food introductions among Asian infants. This study aimed to explore whether nuts can be safely introduced early and regularly from infancy for Asian eczema infants potentially sensitized at a high risk of a food allergy.

## 2. Materials and Methods

This was a retrospective cohort study in the tertiary allergy center in Tokyo, Japan. We examined the infants who had early-onset eczema before 6 months of age and received the instruction of early introduction of hen’s eggs, milk, wheat, peanuts, and tree nuts by certified allergists from August 2020 to February 2021 using medical chart data. The Ethics Committee of our center had approved the study (Reference Number 2023-095). Informed consent was obtained through the opt-out form on the website. Eczema ascertained based on UK Working Party Criteria for the Definition of Atopic Dermatitis. The disease severity of eczema at the time of inclusion was assessed by the Investigator’s Global Assessment (IGA), Patient-Oriented Eczema Measure (POEM), and Eczema Area and Severity Index (EASI). The following course was evaluated by IGA. Allergenic food introduction was performed in cases of well-controlled eczema. The target foods, including peanuts and tree nuts, were instructed to begin when infants were deemed ready for weaning. An instruction example is referred to the guidelines for peanut allergy prevention in the US [17] and given below; they were instructed to mix approximately 0.2–0.5 g of smooth paste or flour into their daily weaning food such as pureed fruit or vegetable. If there are no symptoms after increasing the dosage several times, gradually increase the dose. The frequency of instruction was left to the allergist but was performed as needed, along with checking the intake of weaning food at each visit for eczema. Peanuts and tree nuts were instructed to be consumed in powder or paste to prevent choking. For example, guardians were instructed to purchase already processed paste as a confectionery ingredient. Even if the infants had food allergies, they were included if they could consume a small amount of the allergen. 

Allergen-specific IgE was measured at 6, 12, and 24 months when a food allergy was suspected or when an allergist was considered necessary using a standard ImmunoCAP assay (Thermo Fisher Scientific Inc., Uppsala, Sweden). Sensitization levels were considered ≥0.35 UA/mL. Blood specimens were handled by the contract company (SRL., Inc.; Tokyo, Japan).

Exclusion Criteria were as follows: infants born at less than 37 weeks of gestational age and with low birth weight (<2500 g); those who developed skin lesions such as keratosis or bullous lesions that cannot be assessed adequately or congenital disease with serious complications; and any other infants whom the principal investigator or sub-investigator determined to be unsuitable for the safe conduct of this study. The outcome measure was the consumption of hen’s egg, peanuts, walnuts, and cashew nuts at 12 and 24 months of age and adverse events due to eating allergenic foods. Quantitative parameters were assessed as median, minimum, and maximum values. Fisher exact tests were used to make comparisons between participants with a family history of food allergy. Mann–Whitney’s U test was used for comparisons of total IgE, allergen-specific IgE, TARC, IGA, POEM, and EASI between participants, which are ordinal and continuous variables, respectively.

## 3. Results

**Figure 1:**
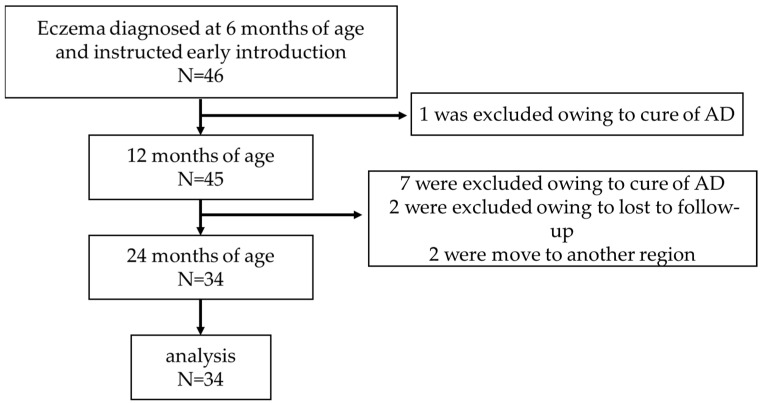
Flow chart of the study.

We observed 46 infants who were instructed for allergenic food introductions (Figure 1). For two years follow-up period, 12 participants were excluded from the analysis owing to the cure of eczema, moved to another region, and lost to follow-up. Finally, we included thirty-four eczema infants in the analysis.

Table 1 shows the background information of infants with eczema. We defined early introduction as a small amount introduced by 24 months of age. A family history of food allergies was more common in the early consumption group for peanuts and walnuts, and cashews were almost equivalent in both groups, although there are no significant differences. Eczema was well controlled in most patients with adequate topical therapy. The severity of eczema at the time of inclusion was almost all mild, with a median EASI of 0, but some were moderate (EASI 12.4, IGA 3). With continued topical therapy, almost all were well controlled, and no worsening of eczema was observed after the start of early introduction. Similarly, there was no difference in the severity of eczema between the early introduction group and the non-early introduction group for peanuts and nuts.

**Figure 2:**
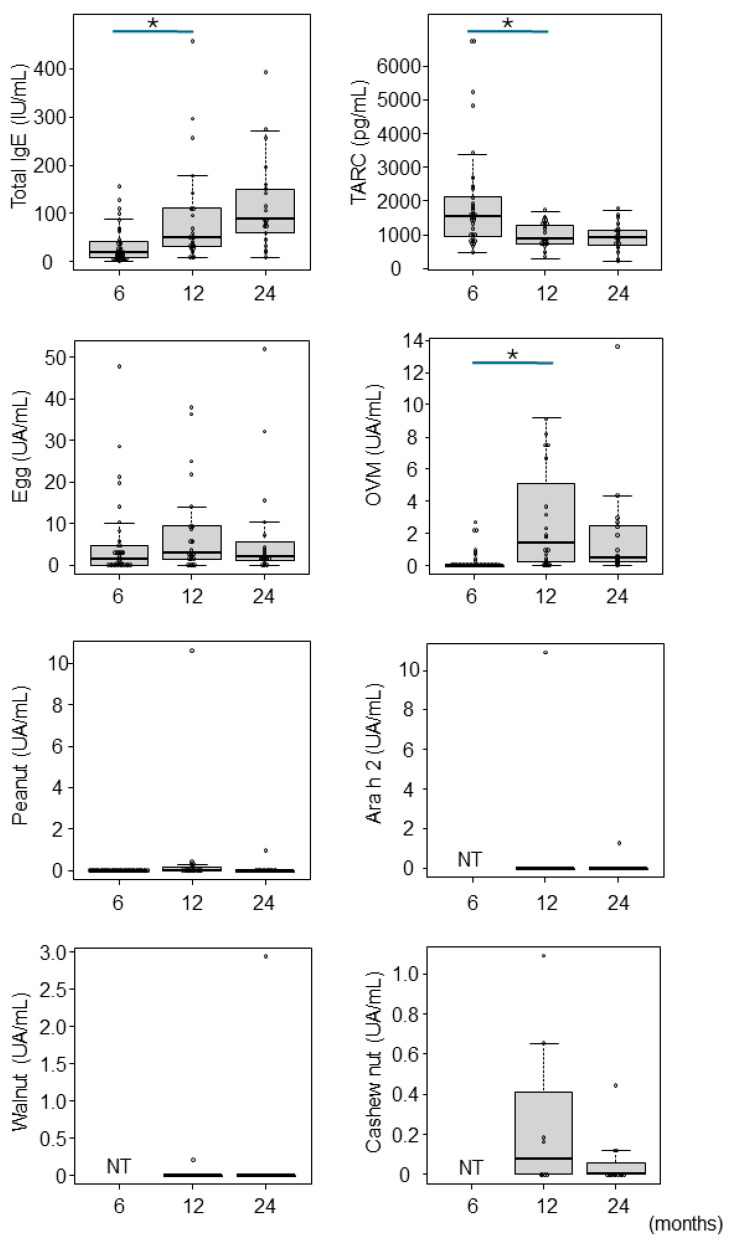
Trend in specific IgE levels and TARC with age by month. Allergen-specific IgE, total IgE, and TARC at 6, 12, and 24 months were investigated for participants whose allergists considered it necessary. Median (IQR) values at 6, 12, and 24 months, respectively, were as follows: total IgE 17.8 (6.98–40.0) IU/mL, 48.9 (31.0–111.0) IU/mL, and 87.2 (59.1–149.0) IU/mL; TARC 1520.5 (964.8–2048) pg/mL, 876.0 (721.0–1287) pg/mL, and 908.5 (706.0–1135) pg/mL; Egg 4.70 (1.40–48.0) UA/mL, 9.41 (3.02–37.9) UA/mL, and 4.89 (2.06–51.9) UA/mL; OVM 0 (0–2.70) UA/mL, 1.37 (0.3–4.41) UA/mL, and 0.5 (0.26–2.50) UA/mL. * indicates statistical significance, *p* < 0.01. NT: not tested, OVM: ovomucoid.

Total IgE levels increased with age in months, with median (IQR) values of 17.8 (6.98–40.0) IU/mL, 48.9 (31.0–111.0) IU/mL, and 87.2 (59.1–149.0) IU/mL at 6, 12, and 24 months, respectively (Figure 2). IgE levels increased significantly from 6 to 12 months (*p* < 0.01). TARC decreased significantly from 6 to 12 months to 1520.5 (964.8–2048) pg/mL and 876.0 (721.0–1287) pg/mL, respectively (*p* < 0.01). The specific IgE of hen’s eggs did not change significantly with age in months. In contrast, ovomucoid significantly increased from 6 to 12 months of age, 0 (0–2.7) UA/mL and 1.37 (0.3–4.41) UA/mL, respectively. At 24 months, it tended to decrease to 0.5 (0.26–2.50) UA/mL, but no significant difference was found. For peanuts, walnuts, and cashews, only a few tests were performed, and most were not sensitized.

**Figure 3:**
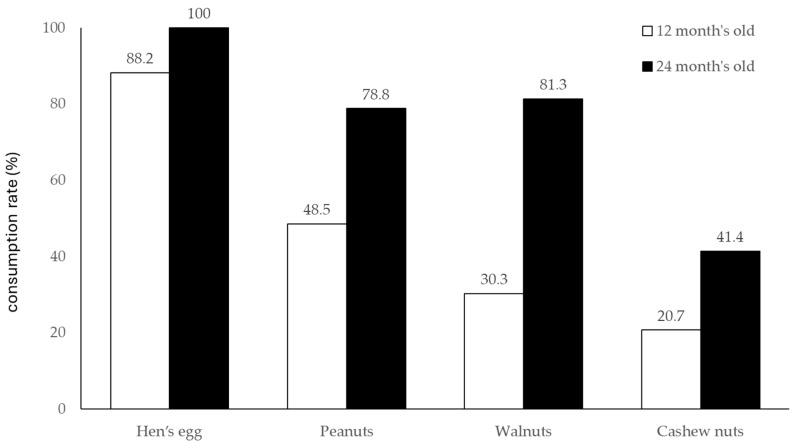
The consumption rate of hen’s egg, peanuts, walnuts, and cashew nuts.

Next, we evaluated the consumption rate of each allergenic food at 12 and 24 months of age (Figure 3). Surprisingly, for hen‘s eggs, the consumption rates were 100% at 24 months. For peanuts and walnuts, the consumption rate was moderate at 12 months (48.5% and 30.3%, respectively), but by 24 months, it had progressed to 78.8% and 81.3%, respectively. In contrast, cashews remained at lower levels than that of other allergens at 20.7% at 12 months and 41.4% at 24 months.

**Figure 4:**
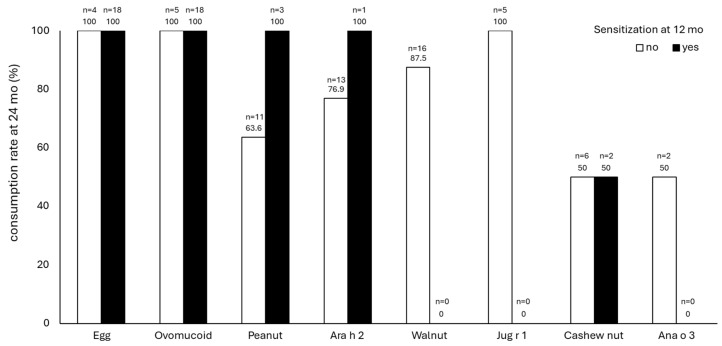
The consumption rate of hen’s egg, peanuts, walnuts, and cashew nuts between sensitized and non-sensitized infants. The white bar shows infants without sanitization, and the black bar shows infants with sensitization.

Figure 4 shows the consumption rate of hen’s egg, peanuts, walnuts, and cashew nuts between sensitized and non-sensitized infants. We investigated whether sensitization in the 12 months of age affected consumption in the following 24 months (Figure 4). For hen’s egg consumption, 100% of both sensitized and non-sensitized children were able to consume it early. Infants without peanut sensitization had an early peanut intake rate of 63.6%. Similarly, infants without Ara h2 sensitization had an early peanut intake rate of 76.9% at 12 months of age. Although there were very few infants with peanut sensitization at 12 months of age, all cases were able to have early peanut intake despite the sensitization. Regarding walnuts, since there were no sensitized children, a comparison between sensitized and non-sensitized children could not be made. A consumption rate of 87.5% was reached for the infants who were not sensitized to walnuts; they did not necessarily achieve 100% consumption, similar to peanuts. For both peanuts and walnuts, the absence of sensitization with allergen components, Ara h 2 and Jug r 1, respectively, in the non-sensitized children improved the consumption rate at 24 months. For cashew nuts, approximately half of both sensitized and non-sensitized children were able to consume them early.

As for the safety of early allergenic food introductions, none of the infants experienced adverse events of choking or aspiration related to the early introduction of any allergenic foods (Table 2). Five infants had immediate reactions and visited the ER for any food reactions, but both were mild and did not require adrenaline administration. All participants had early-onset atopic dermatitis and tended to have a high prevalence of food allergy, but the incidence was not affected by the causative food. Food protein-induced enterocolitis syndrome (FPIES) was caused by egg yolk in all cases.

## 4. Discussion

This study demonstrates the early introduction of tree nuts by powder and paste feeding while maintaining safety in high-risk infants under well-controlled eczema instructed by allergy specialists. This was the first report about early nuts introduction in Asian infants. A pilot study using a mixed powder reported no severe immediate-type allergies in children with or without eczema in the United States. The powder mixture included cashews, almonds, walnuts, and hazelnuts and was administered to 4- to 6-month-old infants for 1 year [18]. In our study, the early consumption of powders and pastes for infants with eczema at 6 months was achieved without adverse events such as allergic symptoms, choking, or aspiration. We believe the results are similar to the US results in terms of the safety and feasibility of the early introduction of tree nuts [18].

We believed that the safe introduction was possible due to guidance from allergy specialists. Currently, procedures for safe introduction by primary care physicians are not well-established. Therefore, there is a need to develop manuals or guidelines to enable safe early nut introduction, not only in allergy clinics but also in general pediatric practices in the future. In addition, we did not observe any difference between infants who were sensitized early and those who were not sensitized in the final consumption rate. We have previously reported that even if a participant is sensitized to a food, tolerance can be achieved by initiating and maintaining the consumption of the food at very low doses [3]. In consideration of these circumstances, we have instructed the early introduction of tree nuts to children, as well as hen’s eggs, milk, and peanuts for eczema infants, regardless of whether participants have IgE sensitization. It is common for infants with eczema to already have IgE sensitization. Therefore, it is generally believed that caregivers are often hesitant to initiate early intake based on the results of IgE testing. We explained that sensitization is not necessarily the same as the development of allergic symptoms and that it is important to consume small amounts of the allergenic food (a non-allergy-reaction-triggering amount). As we instructed early introduction for all infants, we believe that we were able to achieve almost the same consumption rate in the sensitized and non-sensitized infants. There is still little evidence for such an early introduction being associated with tree nut allergies. In the Australian cohort of around 5000 infants, 4.8% had consumed cashews before the age of 12 months. None of them developed cashew allergies at the age of 6 years, while 3.6% of children who had not consumed cashews by 12 months of age developed cashew nut allergies. This report suggests that the early introduction of cashews may be warranted [19]. On the other hand, the early consumption of tree nuts is not widespread enough, even in the US, where it is strongly encouraged. A survey of the general U.S. population revealed that only 26.3% of children consumed nuts before the age of 12 months, and this percentage is significantly lower than that associated with milk and hen’s eggs [20]. In this previous report, the authors mentioned that the rate of peanut introduction is almost equivalent to that of milk and hen’s eggs and that the consumption rate increased over previous reports. In Australia and New Zealand, based on evidence of the importance of early introduction for preventing the development of peanut allergy, academic societies updated their guidelines, and the government was involved and actively promoted dissemination [21]. As a result, the peanut consumption rate in Australia increased dramatically from 28.4% before the guideline revision to 88.6% [22]. It is possible that with the implementation of appropriate measures, the consumption rate of tree nuts could increase, as well as that of peanuts.

Many perspectives on the prevention of food allergy have been attempted, including maternal interventions during the prenatal period [23]. In this study, we focused on participants with early-onset eczema who were at the highest risk of food allergy and examined the feasibility of prevention through early introduction. Of course, some developed food allergies, though they could achieve an extremely high rate of early introductions of hen’s eggs, milk, and wheat. On the other hand, the adoption rate of tree nuts was lower than that of other allergenic foods such as hen’s eggs. There is a need to provide evidence-based and highly feasible food for the early intake of tree nuts. It is widely known that nutrition in the initial days of an infant’s life plays a pivotal role in their growth. The varieties of food consumed during this period also contribute to the development of the immune system by affecting the gut microbiota [24]. The consumption of a variety of foods at the appropriate period without delay has implications beyond the prevention of food allergies. 

There were two infants with FPIES, both caused by egg yolk. In Japan, egg FPIES was the most common, and FPIES caused by egg yolk has been increasing rapidly in recent years [25,26,27,28]. It has been suggested that this is the result of the early consumption of eggs spreading in Japan. However, rice, which is the earliest food consumed as weaning food in Japan, rarely causes FPIES. Globally, rice is one of the most frequent causes of FPIES [29]. Thus, there is no clear evidence that early consumption is involved in the development of FPIES. In this study, we believe that there is little association between the development of egg yolk FPIES and instructions of early introduction.

We had a few limitations: This was a single-center study, but this was the first report to provide data specifically on Asian populations. This was a small pilot study because, in Japan, the Consumer Affairs Agency and the Japan Pediatric Society still recommend abstinence from tree nuts and hard beans until the age of five. Therefore, it is currently impossible to correct big data regarding early nut introductions in Japan. This is the first step in implementing early nut introduction in Asian infants. We will publish the results using national big data [30,31] in the future. The number of participants assessed for sensitization was very small. In clinical practice, participants who could routinely consume did not need to be assessed for sensitization, and blood sampling was not required. Therefore, the consumption rate with or without sensitization might be underestimated. We recommend the early introduction of tiny doses of allergenic foods in all infants, regardless of whether they have sensitization or not. This policy is based on the results of the PETIT Study. We verified through RCT (PETIT Study) that infants with eczema can tolerate early introduction of eggs without allergic symptoms if eczema is well controlled, even if they already have sensitization, thus preventing egg allergy. Some of them may have developed food allergies if they consumed large amounts at once, but they were not diagnosed with food allergies because of the small amount they consumed. Considering the aforementioned, the present study may be overestimating the results. However, the PETIT Study showed that the development of allergies to hen’s eggs can be prevented by starting 50 mg of egg protein consumption at the age of 6 months and gradually increasing the dose to 250 mg of egg protein [3], even in the presence of sensitization to hen’s eggs. We have also previously reported that children who developed food allergies were able to increase their tolerable dose threshold by the continuous consumption of a very small amount of the allergen, such as 1/100 of the initial threshold dose [32,33]. Therefore, we believe that continued consumption, even in small amounts, contributes to tolerance in potentially allergic children.

## 5. Conclusions

In conclusion, each country has various ethnic communities with diverse food cultures. Asian infants with eczema, who are at high risk for food allergy, could be safely introduced to allergenic foods, including tree nuts, early if instructed by an allergist. The early introduction of peanuts and tree nuts was still more challenging in real-world practice in Asia as well as in Western countries.

## Figures and Tables

**Table 1:** The background information of eczema infants.

	Total	Hen’s Egg	Peanuts		*p*-Value	Walnuts		*p*	Cashew Nuts		*p*-Value
Valid response (n)	34	34	33			32			29		
Early introduction		Yes	No	Yes		No	Yes		No	Yes	
Male (%)	22 (64.7)	22 (64.7)	4 (12.1)	17 (51.5)		2 (6.3)	18 (56.3)		8 (27.6)	9 (31.0)	
Mother’s history of FA	7	7	2	5	0.62	1	6	1.0	5	2	0.67
Father’s history of FA	5	5	0	5	0.56	0	5	0.56	2	3	0.62
Older sibling’s FA	1	1	0	1	1.0	0	1	1.0	0	1	0.41
IGA 6mo (median, range)	1 (0–3)	1 (0–3)	1 (0–1)	1 (0–3)	0.09	1 (0–2)	1 (0–3)	0.56	1 (0–3)	1 (0–3)	0.57
IGA 12mo (median, range)	0 (0–2)	0 (0–2)	0.5 (0–2)	0 (0–2)	0.79	0.5 (0–2)	0 (0–2)	0.54	0 (0–2)	1 (0–2)	0.41
IGA 24mo (median, range)	0 (0–2)	0 (0–2)	0.5 (0–2)	0 (0–2)	0.54	0 (0–2)	0 (0–2)	0.67	0 (0–2)	1 (0–2)	0.15
POEM (median, range)	1.5 (0–17)	1.5 (0–17)	3 (0–11)	1 (0–17)	0.80	2.5 (0–11)	2 (0–17)	0.90	2 (0–16)	1 (0–17)	0.62
EASI (median, range)	0 (0–12.4)	0 (0–12.4)	0 (0–4.5)	0 (0–12.4)	0.25	0 (0–4.5)	0 (0–12.4)	0.34	0 (0–7.6)	0 (0–12.4)	0.49

**Table 2:** Allergenic food introductions and related episodes.

	Total	Hen’s Egg	Peanuts		Walnuts		Cashew Nuts	
Valid response (n)	34	34	33		32		29	
Early introduction		Yes	No	Yes	No	Yes	No	Yes
Choking or aspiration	0	0	0	0	0	0	0	0
Episode of allergy reactions related target allergenic foods	0	0	0	0	0	0	0	0
Food hypersensitivity-related ER visit for any foods	5	5	1	4	2	2	3	1
Unscheduled visit regarding any foods	2	2	2	0	1	1	2	0
Adrenaline use	0	0	0	0	0	0	0	0
Episode of any food allergy (%)	8 (23.5)	8 (23.5)	1 (14.3)	7 (26.9)	1 (16.7)	7 (26.9)	4 (23.5)	4 (33.3)
Episode of any FPIES (%)	2 (5.9)	2 (5.9)	0	2 (7.7)	1 (16.7)	0	1 (5.9)	1 (8.3)

FPIES, food protein-induced enterocolitis syndrome.

## Data Availability

Currently, individual participant data sharing is unavailable because the IRB permission is not yet obtained under the Ethical Guidelines for Medical and Biological Research Involving Human Subjects and Personal Information Protection Commission, Japan. The individual participant data sharing will be available after the IRB permission is granted. Any data queries can be emailed to the Primary Investigator, Kiwako Yamamoto-Hanada, at yamamoto-k@ncchd.go.jp.
